# A phase II study of afatinib (BIBW 2992), an irreversible ErbB family blocker, in patients with HER2-positive metastatic breast cancer progressing after trastuzumab

**DOI:** 10.1007/s10549-012-2003-y

**Published:** 2012-03-15

**Authors:** Nancy U. Lin, Eric P. Winer, Duncan Wheatley, Lisa A. Carey, Stephen Houston, David Mendelson, Pamela Munster, Laurie Frakes, Steve Kelly, Agustin A. Garcia, Susan Cleator, Martina Uttenreuther-Fischer, Hilary Jones, Sven Wind, Richard Vinisko, Tamas Hickish

**Affiliations:** 1 Dana-Farber Cancer Institute, 450 Brookline Avenue, Boston, MA 02215 USA; 2 Royal Cornwall Hospital, Truro, UK; 3 University of North Carolina, Chapel Hill, NC USA; 4 Royal Surrey County Hospital, Guildford, UK; 5 Pinnacle Oncology Hematology, Scottsdale, AZ USA; 6 UCSF, Helen Diller Family Comprehensive Cancer Center, San Francisco, CA USA; 7 San Diego Pacific Oncology and Hematology Associates Inc., Encinitas, CA USA; 8 Derriford Hospital, Plymouth, UK; 9 USC Norris Comprehensive Cancer Center and Hospital, Los Angeles, CA USA; 10 Mary’s Hospital, London, UK; 11 Boehringer Ingelheim Pharma GmbH & Co KG, Biberach, Germany; 12 Boehringer Ingelheim Ltd, Bracknell, UK; 13 Boehringer Ingelheim Pharmaceuticals Inc., Ridgefield, NJ USA; 14 Dorset Cancer Centre and Bournemouth Hospital, Bournemouth, UK; 15 Boehringer Ingelheim Pharmaceuticals Inc., Ridgefield, CT USA

**Keywords:** Breast cancer, ErbB1, ErbB2, Human epidermal growth factor receptor, Second-generation small molecule kinase inhibitors, Trastuzumab

## Abstract

Afatinib is an oral, ErbB family blocker, which covalently binds and irreversibly blocks all kinase-competent ErbB family members. This phase II, open-label, single-arm study explored afatinib activity in human epidermal growth factor receptor 2 (HER2)-positive breast cancer patients progressing after trastuzumab treatment. Patients had stage IIIB/IV HER2-positive metastatic breast cancer, with progression following trastuzumab or trastuzumab intolerance and an Eastern Cooperative Oncology Group (ECOG) performance status of 0–2. Patients received 50 mg afatinib once-daily until disease progression. Primary endpoint was objective response rate (Response Evaluation Criteria in Solid Tumors 1.0), with tumor assessments every 8 weeks. Forty-one patients were treated. Patients had received a median of three prior chemotherapy lines (range, 0–15) and 68.3% had received trastuzumab for >1 year. Four patients (10% of 41 treated; 11% of evaluable patients) had partial response. Fifteen patients (37% of 41) had stable disease as best response and 19 (46% of 41) achieved clinical benefit. Median progression-free survival was 15.1 weeks (95% confidence interval [CI]: 8.1–16.7); median overall survival was 61.0 weeks (95% CI: 56.7–not evaluable). Most frequent common terminology criteria for adverse events grade 3 treatment-related adverse events were diarrhea (24.4%) and rash (9.8%). Afatinib monotherapy was associated with promising clinical activity in extensively pretreated HER2-positive breast cancer patients who had progressed following trastuzumab treatment.

**Electronic supplementary material:**

The online version of this article (doi:10.1007/s10549-012-2003-y) contains supplementary material, which is available to authorized users.

## Introduction

Hyperactivation of the ErbB signaling network has been observed in a variety of malignancies and is associated with tumor cell proliferation and metastasis [1, 2]. The ErbB receptor family consists of four receptor tyrosine kinases: epidermal growth factor receptor (EGFR), also known as human epidermal growth factor receptor (HER)1 or ErbB1, HER2 (*neu*/ErbB2), HER3 (ErbB3), and HER4 (ErbB4) [1]. Increased understanding of the role of the ErbB receptor signaling network in cancer has led to development of various agents designed to specifically target these receptors [3]. Prominent examples include ErbB receptor targeting monoclonal antibodies such as trastuzumab, and small-molecule inhibitors such as gefitinib, erlotinib, and lapatinib.

Trastuzumab, a humanized monoclonal antibody directed against the extracellular domain of the HER2 receptor, is indicated for the treatment of HER2-positive breast cancer (BC) in the adjuvant and metastatic setting [4]. However, both primary and acquired resistance are significant clinical problems [5]. Resistance to trastuzumab has been described to occur through many different mechanisms [6–8]. One such mechanism, molecular plasticity of the ErbB pathway axis (HER reprogramming), is commonly observed; some breast tumors switch to alternative HER2 translation resulting in amino-terminally truncated HER2 fragments (611-CTF) [9] which are no longer recognized by the antibody. In time, trastuzumab may fail to inhibit generation of p95HER2 [10], a proteolytic fragment which lacks the extracellular trastuzumab-binding domain and, when present, correlates with resistance to trastuzumab [11]. Increased expression of ErbB family members and cognate ligands such as EGFR/HER1 and EGF, TGFa, Hb-EGF, and heregulin has been demonstrated as a mechanism for acquired resistance to trastuzumab [12] and long-term trastuzumab exposure of primary resistant breast cancer cells is associated with HER1 reprogramming [13]. Due to the presence of HER reprogramming and other resistance mechanisms in HER2-positive BC, inhibition of more than one member of the ErbB family is expected to improve efficacy in this cancer setting. As such, inhibition of more than one member of the ErbB family may maximize inhibition of ErbB signaling with the potential to improve efficacy of targeted ErbB inhibitors.

Afatinib is a novel, potent, small-molecule tyrosine kinase inhibitor (TKI) which irreversibly and selectively targets the ErbB family of receptors: ErbB1 (EGFR/HER1) (IC_50_ 0.5 nM) and ErbB2 (HER2) (IC_50_ 14 nM) [14]. In vitro studies have shown that afatinib treatment inhibits growth of the trastuzumab-resistant SUM 190 cell line, which over expresses HER2 [15, 16] and shows potent antitumor activity in human xenograft models known to depend on ErbB signaling [17]. Furthermore, afatinib has also shown potent antitumor activity in vivo in SUM 190 xenografts, a HER2-positive but trastuzumab-resistant model known to express large amounts of HER2 and displaying activated EGFR/HER1, HER2, and HER3 [16].

Evidence of clinical activity of afatinib monotherapy has also been demonstrated in phase I dose-escalation studies in advanced solid tumors. Stable disease (SD) was achieved in five of 14 BC patients participating in phase I monotherapy trials for up to 12 weeks (three patients) and up to 24 weeks (two patients) [18–20].

This phase II study was conducted to assess the efficacy and safety of afatinib monotherapy in patients with HER2-positive metastatic BC after progression on trastuzumab.

## Patients and methods

### Study design

This phase II, open-label, single-arm, multicenter trial was conducted in the USA and the UK according to the Declaration of Helsinki and in accordance with the International Conference on Harmonization Harmonized Tripartite Guideline for Good Clinical Practice. Written informed consent was obtained from all participants.

### Treatment

All patients received daily oral doses of afatinib (50 mg/day) until disease progression, undue adverse events (AEs) or withdrawal of consent. Dose reduction to 40 mg/day and consecutively 30 mg/day was required for patients experiencing any Common Terminology Criteria for Adverse Events (CTCAE) version 3.0 grade ≥3 drug-related AEs. Patients with CTCAE grade 3 diarrhea or CTCAE grade 2 diarrhea lasting >7 days despite adequate anti-diarrheal treatment, persistent CTCAE grade ≥2 nausea and/or vomiting despite optimal anti-emetic treatment or persistent CTCAE grade ≥3 rash despite optimal supportive care (including systemic antibiotics), were also dose-reduced. Patients with ≥20% decrease in left ventricular ejection fraction (LVEF) from baseline were required to discontinue treatment.

### Study population

Adult female patients, aged ≥18 years, with a confirmed diagnosis of stage IIIB or IV HER2-positive metastatic BC (HER2 2+ by immunohistochemistry (IHC) and fluorescence in situ hybridization-positive or HER2 3+ by IHC) and a life expectancy of ≥4 months, were included in this study. Patients were required to have experienced disease progression following trastuzumab treatment and/or standard chemotherapy in conjunction with trastuzumab. Patients with an Eastern Cooperative Oncology Group (ECOG) performance score of 0–2 and measurable disease according to Response Evaluation Criteria in Solid Tumors (RECIST) version 1.0 were included. All patients were required to have a normal hematological profile and adequate liver, kidney, and bone marrow function, and to have recovered from any therapy-related toxicity from previous chemo-, hormone-, immuno- or radiotherapies.

Patients with active infectious disease, gastrointestinal disorders that may interfere with the absorption of the study drug, chronic diarrhea or serious illness or concomitant non-oncological disease considered incompatible with study participation by the investigator, or active/symptomatic brain metastases were excluded. Patients receiving treatment with other investigational drugs, chemo-, hormone-, immuno- or radiotherapy within 4 weeks before the start of the study (2 weeks for trastuzumab) and patients who had received previous treatment with an EGFR/HER1- or HER2-inhibiting agent (except trastuzumab) were also excluded.

### Concomitant medications

Concomitant medications or therapy to provide adequate care could be given as clinically necessary, although megestrol acetate and hormonal ablative therapy such as leuprolide were excluded. Proactive management of diarrhea, nausea, vomiting, and rash/acne, was encouraged using appropriate medications in accordance with the recommendations for the treatment of AEs provided in the clinical trial protocol. Treatment with chemo-, hormone-, immuno- or radiotherapy was not permitted, with the exception of bisphosphonates and palliative radiotherapy to non-target lesions.

### Efficacy assessments

Tumor assessments were performed after every 8 weeks of treatment throughout the trial. The primary endpoint in this trial was objective response [complete response (CR) and partial response (PR)] as determined by RECIST version 1.0 [21]. Secondary outcome measures included time to progression, progression-free survival (PFS), overall survival (OS), time to objective response, and duration of objective response. In addition, quality of life (QOL) was measured using the European Organization for Research and Treatment-Quality of Life Questionnaire (EORTC QLQ-C30).

### Safety and tolerability assessments

The incidence and severity of AEs according to National Cancer Institute CTCAE version 3.0 were used to assess safety. Physical examination, vital signs, laboratory examinations, and cardiac function were regularly monitored. Changes in the selected laboratory parameters were defined as possibly clinically significant in case of a worsening from baseline of ≥2 CTCAE grades.

### Pharmacokinetic assessments

For quantification of afatinib plasma concentrations, 5 ml of venous blood was collected on day 1 of course 1 and day 15 of course 2: pre-dose, 1, 2, and 3 h after drug administration. In addition, a voluntary pharmacokinetic (PK) sample could be taken within the time frame of 4–24 h after afatinib administration. Additional pre-dose plasma samples were taken on day 15 of course 1 and on day 1 of course 2 and all subsequent courses. Afatinib drug concentrations were determined by a validated high performance liquid chromatography–mass spectroscopy (HPLC–MS/MS) assay.

### Statistical analyses

The analyses in this study were descriptive and exploratory. The efficacy analysis included all patients who received at least one dose of afatinib; in addition, objective response analyses were based on all patients with evaluable tumor measurements. Exact 95% Clopper–Pearson confidence intervals (CIs) were calculated for the proportion of patients showing an objective response to treatment (primary efficacy outcome). Similar point estimates and exact CIs were calculated for CR, PR, and SD. PFS, OS, and time to objective response was estimated from Kaplan–Meier curves. The safety analysis included all patients who received at least one dose of trial medication. Safety findings were summarized using descriptive statistics.

## Results

### Patient population

This study was conducted at six sites in the USA and six sites in the UK between November 2006 and August 2009. In total 52 patients were screened, and 41 patients (21 from the USA; 20 from the UK) received trial treatment with afatinib. Baseline demographics for treated patients are presented in Table 1. Ninety-three percent of patients had an ECOG performance status of 0–1, and the majority (92.7%) had undergone prior surgery. All patients had received prior trastuzumab (as required by the protocol), with 68.3% of patients receiving this treatment for more than 1 year (range: 23–396 weeks). The median number of prior chemotherapy regimens was three. Patients’ best response to trastuzumab is shown in Table 1. The most common sites of metastatic disease were lymph nodes, liver, bone, and lung.

**Table 1:** Baseline demographics (treated patients)

	Afatinib (*n* = 41)
Age (years), median (range)	54 (30–86)
ECOG performance status, *n* (%)	
0	24 (59)
1	14 (34)
2	3 (7)
Progesterone receptor-positive, *n* (%)	12 (29)
Estrogen receptor-positive, *n* (%)	20 (49)
Duration of prior trastuzumab (months), *n* (%)	
≤6	3 (7.3)
6–12	10 (24.4)
12–36	20 (48.8)
>36	8 (19.5)
Best response to trastuzumab, *n* (%)	
Complete response	2 (4.9)
Partial response	13 (31.7)
Stable disease	13 (31.7)
Progressive disease	9 (22.0)
Unknown	2 (4.9)
Not applicable	2 (4.9)
Number of prior chemotherapies	
Median	3
Range	0–15
Other prior therapies; *n* (%)	
Hormone	24 (59)
Radiotherapy	32 (78)
Surgery	38 (93)
Immunotherapy	23 (56)

*ECOG* Eastern Cooperative Oncology Group

For the 41 patients that received at least one dose of afatinib, the mean treatment time on afatinib was 99 days. The majority of patients (73.2%) discontinued due to disease progression; nine (22.0%) discontinued due to AEs and two (4.9%) discontinued for other reasons. Twenty patients (48.8%) required dose reduction to 40 mg, and six patients (14.6%) had a further reduction from 40 to 30 mg.

### Antitumor activity

Of the 41 patients treated with afatinib, 35 patients were evaluable for objective response (Table 2). Six patients were not evaluable for response as no baseline or post-baseline imaging measurements were available, but were included in the denominator for response and efficacy assessments. Four patients (10% of 41 patients; 11% of 35 patients evaluable for objective response based on tumor measurement) achieved a PR and no CRs were observed. Three patients had a PR after 8 weeks while one patient had a PR after 16 weeks. The median (range) duration of PR was 12.0 (7.4–56.1) weeks. In one patient, a 30-year old white female with poorly differentiated infiltrating ductal breast carcinoma and lung metastases, PR was maintained for 56 weeks (Table 2) and the duration of overall clinical benefit in this patient was 64 weeks at which time the patient developed a new lesion. An additional 15 patients (37% of 41 patients; 43% of 35 patients) had SD of whom eight patients achieved SD for >4 months and three patients achieved SD for 6–12 months. The maximum duration of SD was 32 weeks.

**Table 2:** Best response according to RECIST criteria

Overall investigator assessment (best response)	Response^a^			Median duration, weeks (range)
	*n*	% All treated patients (*n* = 41)	% Evaluable patients (*n* = 35)	
Clinical benefit (CR + PR + SD)	19	46	54	17.1 (7.3–64.0)
PR	4	10	11	12.0 (7.4–56.1)
SD	15	37	43	–
Progressive disease	16	39	46	–

*RECIST* Response Evaluation Criteria in Solid Tumors, *CR* complete response, *PR* partial response, *SD* stable disease

^a^Six patients were not evaluable for response as no post-baseline imaging measurements were available

**Fig. 1:**
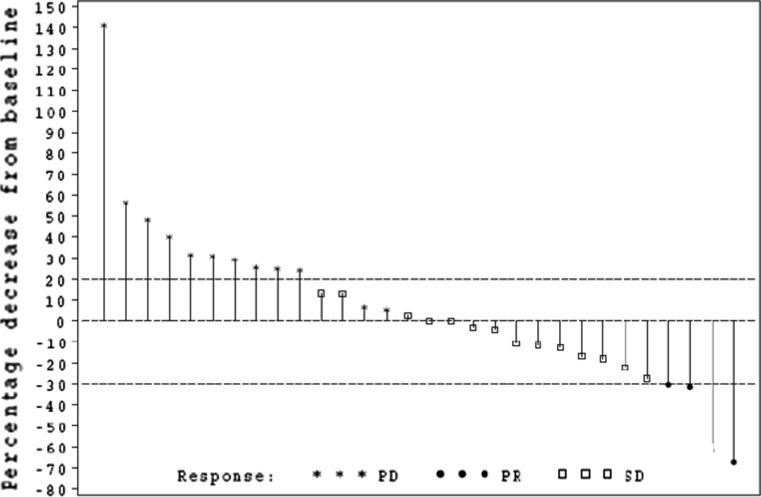
Best RECIST response*. *30 patients had available tumor diameter measurements; five patients had no tumor diameter measurements available (two patients had fewer lesions measured than at baseline, three patients had no post-baseline measurements available, but new lesions documented). *RECIST* Response Evaluation Criteria in Solid Tumors

Overall, 19 patients (46% of 41 patients) were classed as having achieved clinical benefit (CR or PR or SD) with a median (range) duration of clinical benefit of 17.1 (7.3–64.0) weeks. A total of 30 patients had available tumor diameter measurements as depicted in the waterfall plot (Fig. 1). Of the 15 evaluable patients with SD, nine patients demonstrated a decrease in tumor size which did not reach the 30% threshold for PR.

**Fig. 2:**
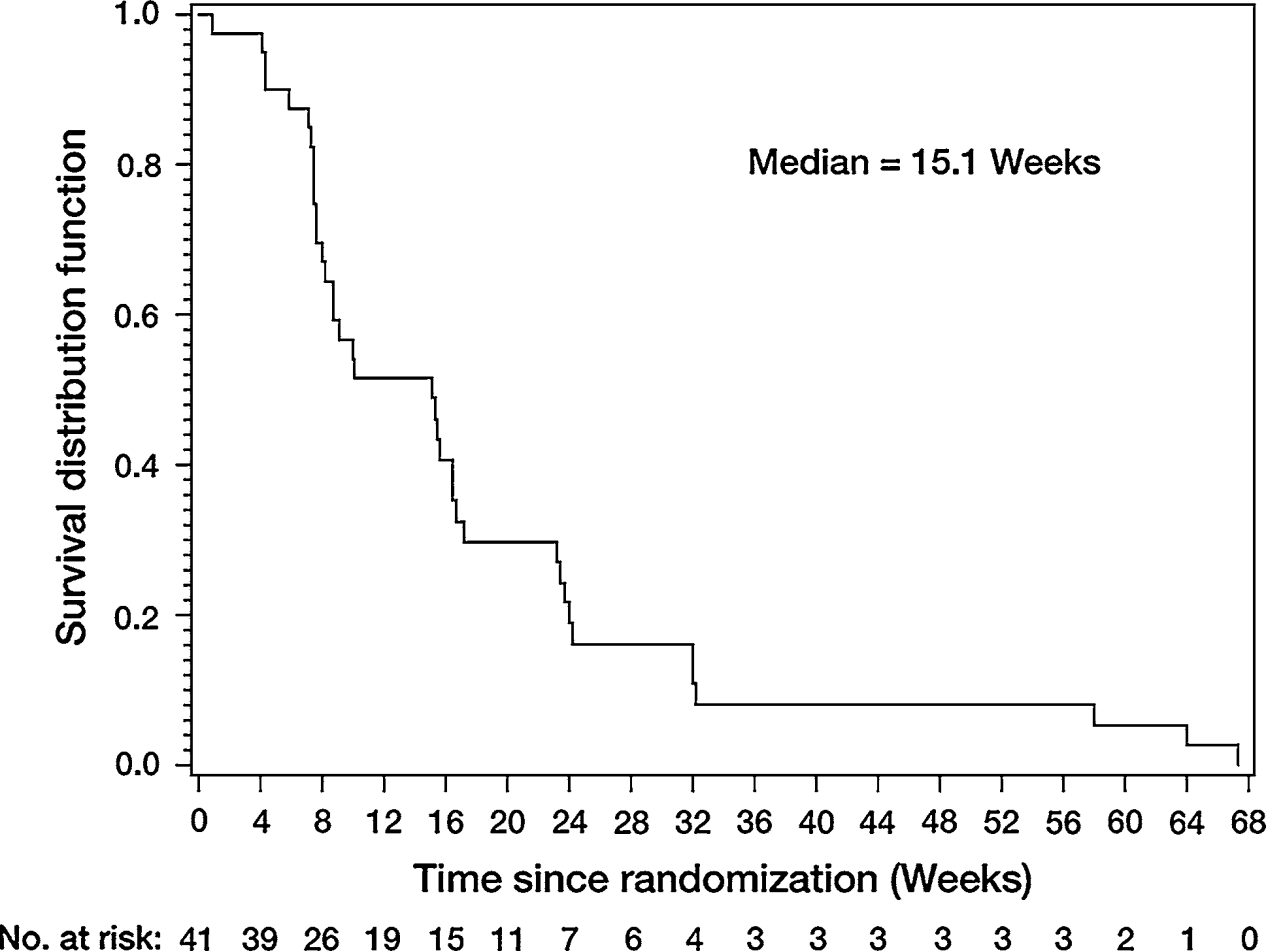
Progression-free survival (treated set)

**Fig. 3:**
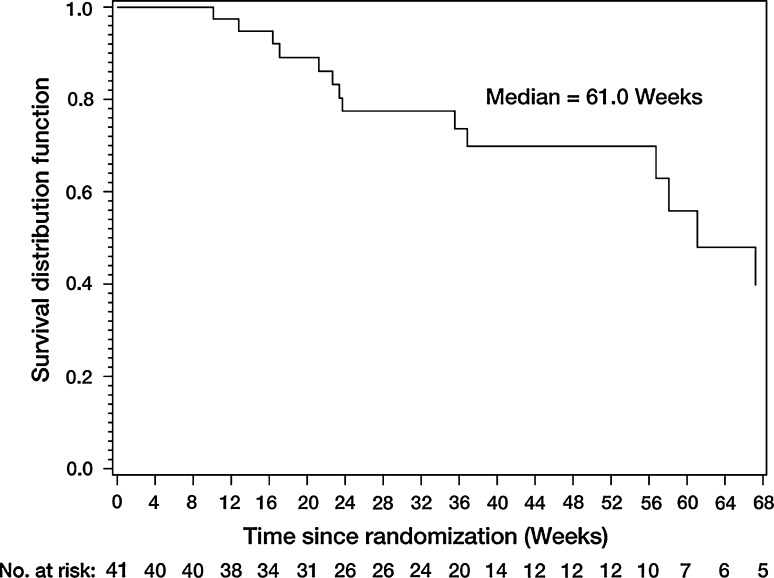
Overall survival (treated set)

In the total population the median PFS was 15.1 weeks (Fig. 2; 95% CI: 8.1–16.7) and a total of 14 patients were known to have died during, or after, the study. The median OS was 61.0 weeks (95% CI: 56.7–not evaluable) (Fig. 3).

### Safety and tolerability

Forty patients (97.3%) experienced treatment-related AEs (according to CTCAE version 3.0) during treatment. The most common treatment-related AEs were diarrhea (90.2%), rash (65.9%), and fatigue (41.5%). Most AEs reported were mild to moderate in severity (CTCAE grade 1 or 2). Treatment-related AEs occurring in more than 5% of patients, or with a CTCAE grade 3, are shown in Table 3.

**Table 3:** Drug-related adverse events according to CTCAE grade (total frequency >5% or grade ≥3), sorted according to frequency

*N* (%)	All treatment-related AEs	CTCAE grade^a^		
		Grade 1	Grade 2	Grade 3
Number of patients	41	41	41	41
Total with related AEs	40 (97.6)	3 (7.3)	17 (41.5)	20 (48.8)
Diarrhea	37 (90.2)	18 (43.9)	9 (22.0)	10 (24.4)
Rash	27 (65.9)	7 (17.1)	16 (39.0)	4 (9.8)
Fatigue	17 (41.5)	10 (24.4)	7 (17.1)	0 (0.0)
Nausea	16 (39.0)	9 (22.0)	6 (14.6)	1 (2.4)
Stomatitis	15 (36.6)	6 (14.6)	6 (14.6)	3 (7.3)
Vomiting	14 (34.1)	5 (12.2)	6 (14.6)	3 (7.3)
Dry mouth	8 (19.5)	8 (19.5)	0 (0.0)	0 (0.0)
Anorexia	7 (17.1)	3 (7.3)	4 (9.8)	0 (0.0)
Dermatitis acneiform	7 (17.1)	4 (9.8)	3 (7.3)	0 (0.0)
Epistaxis	7 (17.1)	7 (17.1)	0 (0.0)	0 (0.0)
Dry skin	6 (14.6)	5 (12.2)	1 (2.4)	0 (0.0)
Dysgeusia	6 (14.6)	6 (14.6)	0 (0.0)	0 (0.0)
Mucosal inflammation	5 (12.2)	3 (7.3)	1 (2.4)	1 (2.4)
Oral pain	5 (12.2)	4 (9.8)	1 (2.4)	0 (0.0)
Pruritus	5 (12.2)	4 (9.8)	0 (0.0)	1 (2.4)
Skin fissures	5 (12.2)	2 (4.9)	2 (4.9)	1 (2.4)
Anemia	4 (9.8)	1 (2.4)	2 (4.9)	1 (2.4)
Dehydration	4 (9.8)	0 (0.0)	2 (4.9)	2 (4.9)
Alopecia	3 (7.3)	3 (7.3)	0 (0.0)	0 (0.0)
Dyspnea	3 (7.3)	2 (4.9)	1 (2.4)	0 (0.0)
Headache	3 (7.3)	3 (7.3)	0 (0.0)	0 (0.0)
Keratoconjunctivitis sicca	3 (7.3)	2 (4.9)	1 (2.4)	0 (0.0)
Pain in extremity	3 (7.3)	1 (2.4)	2 (4.9)	0 (0.0)
Skin reaction	2 (4.9)	0 (0.0)	1 (2.4)	1 (2.4)
Hyponatremia	1 (2.4)	0 (0.0)	0 (0.0)	1 (2.4)
Leukocytoclastic vasculitis	1 (2.4)	0 (0.0)	0 (0.0)	1 (2.4)
Palmar plantar erythrodysaesthesia syndrome	1 (2.4)	0 (0.0)	0 (0.0)	1 (2.4)
Vertigo	1 (2.4)	0 (0.0)	0 (0.0)	1 (2.4)

*CTCAE* common terminology criteria for adverse events, *AE* adverse event

^a^No drug related CTCAE grade 4 events were reported

A total of five patients (12%) experienced serious treatment-related AEs: one patient experienced CTCAE grade 3 dehydration and hyponatremia, one patient experienced CTCAE grade 3 dehydration, diarrhea, and nausea, one patient experienced CTCAE grade 3 nausea and vomiting, one patient experienced CTCAE grade 2 vomiting and one patient experienced CTCAE grade 3 rash, a biopsy of which showed features of a leukocytoclastic vasculitis.

No clinically significant changes indicative of an adverse effect of afatinib were observed in LVEF, or electrocardiogram, or for any laboratory parameter assessed including blood chemistry and liver function tests.

### Other endpoints

ECOG performance score improved during the course of treatment in 24 (60.0%) patients and remained stable in 15 (37.5%) patients; only one (2.5%) patient had an ECOG status that deteriorated. Thirty-nine of the 41 patients were assessed for QOL. Of these, the overall QOL score improved in 15 (38.5%) patients, remained stable in 18 (46.2%) patients and deteriorated in five (12.8%) patients, based on the best post-baseline response. One patient had only baseline data available and was classified as missing. Notable changes in the QOL functional and symptom scales included improvements in the domains of fatigue, insomnia, and pain, with approximately half of all patients recording an improvement in QOL in these areas; QOL with respect to the occurrence of diarrhea was noted as deteriorating in 12 of 39 (30.8%) patients.

### Pharmacokinetics

The starting dose used in this study was 50 mg afatinib; dose reductions to 40 or 30 mg were permitted during treatment and therefore no single-dose PK data were available for these dose levels. Four patients who provided data for PK analysis received a dose of 30 mg afatinib. As such, only PK data from patients receiving 50 or 40 mg afatinib were considered in the PK analysis.

Afatinib plasma concentrations were in steady-state at day 15 (obtained by visual inspection). Steady-state may have been attained earlier, but no PK sampling was performed between days 1 and 15. Trough concentrations were higher in patients receiving afatinib 50 mg compared to those receiving 40 mg afatinib (Supplementary Table S1) and remained stable over the observation period. The overall variability in afatinib trough plasma concentrations was moderate to high (gCV values of 30.7–75.1%; Supplementary Table S1).

## Discussion

This phase II study was designed to assess the efficacy and safety of afatinib in patients with HER2-positive metastatic BC after failure of treatment with trastuzumab. Afatinib demonstrated antitumor activity in this patient group with confirmed PRs and durable SD: 19 patients achieved clinical benefit (46% of 41 patients), with four patients (10% of 41 patients) achieving a PR. A total of 15 patients maintained SD with nine of these patients demonstrating a reduction in tumor size. The median duration of clinical benefit was 17.1 weeks. Median PFS was 15.1 weeks and median OS was 61.0 weeks. This was a heavily pretreated population; the median number of prior chemotherapy regimens was three and nearly 70% of patients had received prior trastuzumab therapy for ≥12 months, with 36.6% of these patients reporting a CR or PR on trastuzumab. With the caveat that this study was a single-arm, phase II monotherapy trial with a limited number of patients, these results are interesting when compared to those obtained upon dual HER2-blockade with lapatinib and trastuzumab in a randomized phase II trial in a similar population [22]. Here, the reported PFS was 12.0 weeks and OS was 51.6 weeks for the combination compared to 8.1 and 39.0 weeks for lapatinib alone. No significant difference was observed in overall response rate for the combination arm compared to the monotherapy arm (10.3 vs. 6.9%; *P* = 0.46). Data reported here with afatinib confirm preliminary results from ongoing studies showing that resistance to trastuzumab can be circumvented by EGFR/HER1 and HER2 targeted TKI therapy. In addition to the antitumor effects of afatinib, ECOG status and QOL assessments also improved during the study, further supporting the benefits of treatment.

Although cross-trial comparisons are limited by methodological differences in study design, patient population and other clinical factors, the results reported in this monotherapy trial are encouraging in relation to effects of other HER2 and EGFR/HER1 inhibitors in similar populations of patients with HER2-positive metastatic BC. Lapatinib, a reversible EGFR/HER1 and HER2 TKI, has shown modest activity in patients with HER2-positive metastatic BC (*n* = 104) who have received ≥3 lines of prior anticancer therapy and trastuzumab therapy [23]. Burstein and colleagues reported an overall response rate of 4.3% (95% CI: 1.6–9.1), clinical benefit rate of 5.7% (95% CI: 2.5–10.9), and median PFS of 9.1 weeks (95% CI: 8.0–13.6) with lapatinib monotherapy. With neratinib, an irreversible inhibitor of EGFR/HER1 and HER2, an objective response rate of 24% (95% CI: 14–36) with a median PFS of 22.3 weeks was reported in patients with HER2-positive metastatic BC (*n* = 66) who have received prior trastuzumab [24].

As noted previously, more recently the effects of dual HER2-blockade have been investigated by Blackwell and colleagues [22]. A phase I trial of assessing the safety and preliminary antitumor activity of afatinib in combination with trastuzumab in patients with advanced HER2-positive BC is ongoing.

Afatinib showed a manageable side effect profile in this study. Similar to previous studies with afatinib, the most frequently reported AEs were diarrhea and rash [18, 19, 25, 26]. These AEs were generally manageable with appropriate treatment pause, supportive care, and dose reductions. Early and pre-emptive management of diarrhea is crucial to prevent potential complications. Most AEs reported with afatinib were mild to moderate in severity (CTCAE grade 1 or 2); no CTCAE grade 4 treatment-related AEs occurred in this study and no treatment-related deaths were reported. In general, the tolerability profile of afatinib reported here was similar to that of EGFR TKIs and consistent with that expected with this class of agent [27].

Cardiotoxicity is a potential issue for patients treated with trastuzumab and it has been suggested to be a class effect for HER2-targeting agents. Therefore, LVEF monitoring is conducted in all afatinib clinical trials. No significant cardiac safety issues were observed in this study.

The PK characteristics of afatinib have previously been evaluated in phase I dose escalation studies, performed in cancer patients and have indicated oral bioavailability and moderately fast absorption [19, 25, 26, 28–30]. Following oral administration, maximum concentrations of afatinib (*C*_max_) are generally observed 1–6 h (*t*_max_) post-dose, either after single dose or at steady-state [19, 25, 26, 28]; steady-state is typically reached within 8 days after first administration. The PK findings reported here in patients with advanced metastatic BC appear similar. In this study there was no detectable change (increase or decrease) in afatinib plasma concentrations with long-term treatment.

In summary, treatment with afatinib showed promising clinical activity in HER2-positive BC patients who had progressed following treatment with trastuzumab. Afatinib has a manageable AE profile with frequent cutaneous AEs and diarrhea. Further clinical trials of afatinib in this patient population are planned.

## Electronic supplementary material

Below is the link to the electronic supplementary material.

Supplementary material 1 (DOCX 13 kb)
