# Supplementary material for: A phase II study of afatinib (BIBW 2992), an irreversible ErbB family blocker, in patients with HER2-positive metastatic breast cancer progressing after trastuzumab
Source: Breast Cancer Res Treat. 2012 Mar 15;133(3):1057–65. doi: 10.1007/s10549-012-2003-y (PMC3387495; doi:10.1007/s10549-012-2003-y)
Supplement: Supplementary file 1 — Supplementary material 1 (DOCX 13 kb) [file 10549_2012_2003_MOESM1_ESM.docx]

**Supplementary Material**

**Table S1:** Comparison of afatinib trough plasma concentrations for patients receiving 40 mg or 50 mg afatinib on Day 15/Cycle 1 (C_pre,ss,15_), Day 1/Cycle 2 (C_pre,ss,29_) and on Day 15/Cycle 2 (C_pre,ss,43_)

| **Afatinib** | **40 mg** | | | **50 mg** | | |
| --- | --- | --- | --- | --- | --- | --- |
|  | N | gMean | gCV [%] | N | gMean | gCV [%] |
| **C_pre,ss,15_** (ng/mL) | – | – | – | 21 | 39.6 | 75.1 |
| **C_pre,ss,29_** (ng/mL) | 7 | 24.6 | 30.7 | 18 | 32.5 | 70.8 |
| **C_pre,ss,43_** (ng/mL) | 9 | 24.9 | 50.7 | 17 | 31.4 | 73.8 |
